# Comparison of bulk electron density and voxel‐based electron density treatment planning

**DOI:** 10.1120/jacmp.v12i4.3522

**Published:** 2011-11-15

**Authors:** Aliaksandr Karotki, Katherine Mah, Gert Meijer, Michael Meltsner

**Affiliations:** ^1^ Department of Medical Physics Odette Cancer Center, Sunnybrook Health Sciences Center Toronto ON Canada; ^2^ Department of Radiation Oncology University of Toronto Toronto ON Canada; ^3^ Department of Radiotherapy Catharina Hospital Eindhoven The Netherlands; ^4^ Philips Radiation Oncology Systems Fitchburg WI USA

**Keywords:** magnetic resonance imaging simulation, bulk density, treatment planning, head and neck cancer, intensity‐modulated radiation therapy

## Abstract

The use of magnetic resonance imaging (MRI) alone for radiation planning is limited by the lack of electron density for dose calculations. The purpose of this work is to evaluate the dosimetric accuracy of using bulk electron density as a substitute for computed tomography (CT)‐derived electron density in intensity‐modulated radiation therapy (IMRT) treatment planning of head and neck (HN) cancers. Ten clinically‐approved, CT‐based IMRT treatment plans of HN cancer were used for this study. Three dose distributions were calculated and compared for each treatment plan. The first calculation used CT‐derived density and was assumed to be the most accurate. The second calculation used a homogeneous patient density of $1\;g/{\text{cm}}^{3}$. For the third dose calculation, bone and air cavities were contoured and assigned a uniform density of $1.5\;g/{\text{cm}}^{3}$ and $0\;g/{\text{cm}}^{3}$, respectively. The remaining tissues were assigned a density of $1\;g/{\text{cm}}^{3}$. The use of homogeneous anatomy resulted in up to 4%–5% deviations in dose distribution as compared to CT‐derived electron density calculations. Assigning bulk density to bone and air cavities significantly improved the accuracy of the dose calculations. All parameters used to describe planning target volume coverage were within 2% of calculations based on CT‐derived density. For organs at risk, most of the parameters were within 2%, with the few exceptions located in low‐dose regions. The data presented here show that if bone and air cavities are overridden with the proper density, it is feasible to use a bulk electron density approach for accurate dose calculation in IMRT treatment planning of HN cancers. This may overcome the problem of the lack of electron density information should MRI‐only simulation be performed.

PACS number: 87.55.D‐

## I. INTRODUCTION

Due to its superior soft tissue contrast, magnetic resonance imaging (MRI) is considered as the imaging modality of choice for targeting of various sites treated with radiation therapy.^(^ ^1^ ^–^ ^3^ ^)^ Nevertheless, MRI is usually not used as a stand‐alone radiation therapy simulator for many reasons.^(^ ^4^ ^,^ ^5^ ^)^ It does not provide electron density information for treatment planning calculations. There is no straightforward way of generating digitally reconstructed radiographs.^(^ ^6^ ^)^ Geometric distortions can be significant, with a magnitude of 1 to 2 cm at the periphery of large fields of view.^(^ ^7^ ^,^ ^8^ ^)^ Hence, the use of MRI in radiation therapy treatment planning is usually accompanied by a computed tomography (CT) scan. MRI and CT images are coregistered. MRI images are used for contouring, while CT images are used for dose calculation and accurate spatial information. MRI simulation without CT is desirable, as it would remove uncertainties introduced by image fusion, simplify logistics, and eliminate X‐ray dose to the patients from CT.

Accurate information on electron density is considered to be crucial in radiation therapy dose calculations when significant inhomogeneities are present. The electron density within a patient ranges from nearly zero (airways) to almost twice that of water (cortical bone), with an average near water density. CT provides voxel‐based electron density, which is used for inhomogeneity corrections by treatment planning systems. In the absence of CT‐derived electron density (i.e., MRI alone), assigning bulk electron density to various tissues may still result in accurate dose determination.

There have been several studies comparing dosimetry of treatment plans based on CT‐derived electron density to those based on bulk electron density.^(^ ^9^ ^–^ ^16^ ^)^ To date, most of them have been limited to brain^(^ ^9^ ^–^ ^12^ ^)^ and pelvis,^(^ ^13^ ^,^ ^14^ ^)^ where there are relatively few inhomogeneities. The patient is either assumed to be homogeneous and water‐equivalent, or bone is contoured and assigned an appropriate density while the rest of the patient is assumed to be water‐equivalent. In these sites, the use of bulk electron density is feasible with dosimetric results similar to plans based on voxel‐by‐voxel density provided by CT. Recently, more complicated sites like thorax^(^ ^15^ ^)^ and head and neck^(^ ^15^ ^,^ ^16^ ^)^ have also attracted attention. The lung and air cavities are contoured in addition to bone and assigned specific density. Again, the results are encouraging. We would like to investigate application of this approach to the head and neck (HN) region, which is known to benefit significantly from MRI imaging. HN includes heterogeneities ranging from airways to cortical bone. It contains a large number of organs at risk (OAR) and, consequently, demands a high level of accuracy in the dose calculations.

To improve accuracy, it is preferable to segment as many tissue types as possible and assign them appropriate density. Unfortunately, such approach is not practical. A more realistic approach is to contour limited number of tissues, whose segmentation can potentially be automated. Intensity‐modulated radiation therapy (IMRT) is becoming the standard of practice for HN cancers. With multiple beams used in IMRT, the impact of various heterogeneities encountered in the HN region on the overall dose may be reduced. Hence potentially high dosimetric accuracy may be achieved by using a limited number of bulk densities.

The purpose of this study was to evaluate the dosimetric accuracy of IMRT treatment planning of HN cancers using three bulk densities: $0\;g/{\text{cm}}^{3}$ for air cavities, $1.5\;g/{\text{cm}}^{3}$ for bone, and $1\;g/{\text{cm}}^{3}$ for all other tissues. In this study, no MRI images were used for the dose calculation. Instead, CT images with overridden density were used. In this way, the effects of gradient distortion and volumetric uncertainties associated with MRI could be eliminated, while allowing a direct comparison of the dose distribution with and without bulk densities. We also calculated dose distribution using homogeneous water‐equivalent anatomy to assess the need for bone and air cavities bulk density overrides.

## II. MATERIALS AND METHODS

Ten clinically approved IMRT treatment plans for HN cancer were chosen (Table 1) to encompass various scenarios in IMRT treatment planning. The plans differed by the site being treated, the number of beams (5 to 8) and beam configurations (all coplanar or not). Primary planning target volume (PTV) referred to the PTV associated with gross tumor volume, while secondary PTV referred to the PTV associated with the subclinical component of the disease excluding the primary PTV. The energy of all the beams was 6 MV.

**Table 1:** Planning data for ten HN IMRT patients.

		*Number of Beams*		*Prescription, Gy/#fractions*	
*Patient*	*Site*	*Coplanar*	*Noncoplanar*	*Primary PTV*	*Secondary PTV*
#1	buccal mucosa	6	1	70/33	56/33
#2	hypopharynx	7	1	70/33	59.4/33
#3	tongue	7	1	70/33	54/33
#4	jugular bulb	6	0	35/15	N/A
#5	chin	7	0	66/33	60/33
#6	base of tongue	7	0	70/33	59.4/33
#7	right orbit	4	1	60/30	54/30
#8	nasopharynx	7	0	70/33	59.4/33
#9	base of tongue	7	0	70/35	56/35
#10	hypopharynx	7	0	70/33	56/33

The dose calculations were done in the ${\text{Pinnacle}}^{3}$ treatment planning system Pinnacle 8.1x (Philips Medical Systems, Fitchburg, WI) using the Collapsed Cone Convolution Superposition algorithm. Three dose distributions were calculated for every treatment plan. All three calculations utilized identical beam parameters. The first dose distribution was calculated using CT‐derived electron density. It is referred to as “heterogeneous”. The second dose distribution assumed a uniform patient density of $1\;g/{\text{cm}}^{3}$ and is referred to as “homogeneous”. For the third dose distribution, bone and air cavities were contoured and assigned density of $1.5\;g/{\text{cm}}^{3}$ and $0\;g/{\text{cm}}^{3}$, respectively. The density for the rest of the tissue was set to $1\;g/{\text{cm}}^{3}$. The third distribution is referred to as “bulk density”. The contouring was done with the auto‐threshold tool in Pinnacle. The threshold was set to 200 HU for bone. For air cavities, the threshold minimum was set to −1000 HU and the threshold maximum to −900 HU. In some cases, these thresholds resulted in parts of the immobilization devices being included into the contours, thereby necessitating a manual edit. If the original treatment plan contained density overrides due to implants (e.g., dental fillings) and artifacts, the density overrides were kept the same in all the calculations.

Following calculations, the dose distributions were compared with the help of the following dose‐volume parameters: $D_{\mathrm{mean}}-\text{mean dose delivered to PTV or OAR}$; V95% – volume of the PTV receiving 95% of the prescribed dose; $D_{\min}-\text{minimum point dose delivered to PTV}$; $D_{\min}(2\;\text{cc})-\text{minimum dose}$ delivered to $2\;{\text{cm}}^{3}$ of PTV; $D_{\max}$ – maximum point dose delivered to PTV or OAR; $D_{\max}\left(2\;\text{cc}\right)$ – maximum dose delivered to $2\;{\text{cm}}^{3}$ of PTV or OAR.

## III. RESULTS

Table 2 presents data describing dose coverage of primary and secondary PTVs in ten patients. Patient #4 did not have a secondary PTV. The numbers in the Table are the percentage deviations of the homogeneous and bulk density plans from the heterogeneous distribution.

**Table 2:** Percentage deviation of the various dosimetric parameters describing primary and secondary PTV coverage in homogeneous and bulk density treatment plans from the heterogeneous dose distribution. The dosimetric parameters differing by 2% or more from heterogeneous calculations are shaded in blue.

*Patient*	*#1*	*#2*	*#3*	*#4*	*#5*	*#6*	*#7*	*#8*	*#9*	*#10*
*Primary PTV (Homogeneous Plan)*										
$D_{\mathrm{mean}},\%$	**2.2**	1.0	0.8	**2.0**	1.3	1.2	1.7	1.8	1.1	0.6
V95%	0.0	0.1	0.2	0.2	0.2	0.0	0.0	0.9	0.1	0.0
$D_{\min},\%$	−0.3	0.4	1.4	**2.0**	0.0	0.4	**2.0**	**2.2**	0.1	1.2
$D_{\min}(2\;\text{cc}),\%$	0.4	0.9	1.0	**2.1**	1.0	0.8	1.5	**3.2**	1.1	0.4
$D_{\max},\%$	**4.7**	0.1	**2.1**	**3.3**	0.6	**3.3**	**2.1**	0.8	1.0	0.9
$D_{\max}(2\;\text{cc}),\%$	4.4	0.9	1.4	**2.4**	1.3	**2.0**	**2.2**	**2.0**	1.1	1.1
*Primary PTV (Bulk Density Plan)*										
$D_{\mathrm{mean}},\%$	0.8	0.4	0.4	0.0	0.2	0.1	0.2	0.7	0.4	0.1
V95%	0.0	0.0	−0.1	0.0	0.1	0.0	0.0	0.4	0.0	0.0
$D_{\min},\%$	−0.3	−0.2	0.2	−0.2	0.0	−0.1	0.5	0.8	0.0	0.7
$D_{\min}(2\;\text{cc}),\%$	0.2	0.3	0.3	0.1	0.4	0.1	0.0	0.9	0.4	−0.1
$D_{\max},\%$	0.8	−0.3	0.5	0.8	0.2	1.4	0.6	0.6	0.3	−0.2
$D_{\max}(2\;\text{cc}),\%$	1.1	0.5	0.5	−0.1	0.3	0.4	0.3	0.8	0.4	0.2
*Secondary PTV (Homogeneous Plan)*										
$D_{\mathrm{mean}},\%$	0.8	1.2	1.0		0.2	0.3	1.8	1.5	1.0	0.5
V95%	0.3	0.4	0.6		0.3	0.4	0.4	**2.0**	0.7	0.0
$D_{\min},\%$	−0.4	0.3	1.0		0.6	0.6	1.7	**2.3**	0.9	0.4
$D_{\min}(2\;\text{cc}),\%$	0.2	1.8	1.7		0.5	0.5	**2.1**	1.1	**2.0**	0.1
$D_{\max},\%$	**4.4**	1.0	**2.4**		0.5	**3.8**	1.5	0.6	1.0	0.3
$D_{\max}(2\;\text{cc}),\%$	**2.4**	1.0	1.6		1.3	**2.6**	1.8	1.1	1.2	0.9
*Secondary PTV (Bulk Density Plan)*										
$D_{\mathrm{mean}},\%$	0.4	0.3	0.2		−0.1	−0.4	0.1	0.4	0.1	0.1
V95%	0.2	0.2	0.3		0.2	−0.3	0.1	1.0	0.0	0.0
$D_{\min},\%$	−0.3	0.0	0.5		0.6	0.4	0.2	−0.1	0.4	−0.1
$D_{\min}(2\;\text{cc}),\%$	0.2	0.4	0.4		0.1	−0.3	0.1	0.2	−0.2	0.0
$D_{\max},\%$	0.7	0.7	0.1		0.1	1.6	0.5	0.4	0.4	0.2
$D_{\max}(2\;\text{cc}),\%$	0.5	0.3	0.3		0.0	0.4	0.2	0.3	0.4	0.2

**Figure 1:**
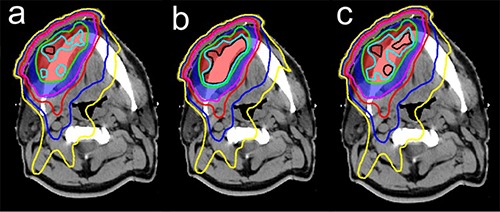
Dose distributions calculated for patient #1 using CT‐derived density (a), homogeneous density (b), and bulk density (c). The red shaded region is primary PTV and the blue is secondary PTV. Primary PTV was prescribed at 7000 cGy, secondary PTV was prescribed at 5600 cGy. The isodose lines shown are 7490 cGy (black), 7350 cGy (light blue), 7000 cGy (green), 5600 cGy (purple), 4900 cGy (red), 3500 cGy (blue), and 2500 cGy (yellow).

In many cases, the dose coverage of the PTV predicted by homogeneous distribution differed substantially from that obtained with CT‐derived density. The dose calculation uncertainty below 2% is often considered insignificant in overall uncertainty of the dose delivery.^(^ ^17^ ^)^ For homogeneous calculations, at least one dose‐evaluation parameter describing PTV coverage differed by 2% or more for seven out of ten patients. The largest deviation was observed for patient #1 where the maximum point dose $\left(D_{\max}\right)$ to primary PTV was 4.7% greater than in heterogeneous case. Note that the clinically significant volume of $2\;{\text{cm}}^{3}$ had a deviation that was still above 4%. Figure 1 shows an example of this discrepancy. The three dose distributions shown are heterogeneous (Fig. 1(a)), homogeneous (Fig. 1(b)), and bulk density (Fig. 1(c)). Since attenuation due to bone is underestimated by the homogeneous plan, the volume of the PTV covered by 7490 cGy is significantly larger than in heterogeneous plan. Assigning bone a density of $1.5\;g/{\text{cm}}^{3}$ in the bulk density plan corrects the problem, reducing maximum point dose deviation from 4.7% to 0.8%.

For the bulk density calculations, all the dosimetric parameters were within 2% of the heterogeneous dose calculations, with most of the parameters being within 1%. Only two patients showed deviations exceeding 1%. In patient #1, maximum dose to $2\;{\text{cm}}^{3}$ of tissue in primary PTV was 1.1% higher than in heterogeneous plan. In patient #6, maximum point dose to both primary and secondary PTV was higher by 1.4% and 1.6%, respectively. Note that in the case of patient #6, the deviation dropped to 0.4% for both PTVs if dose to $2\;{\text{cm}}^{3}$ of tissue was considered.

Table 3 presents data describing dose delivered to OARs. The numbers shown are the percentage deviations of the homogeneous and bulk density plans from the heterogeneous distribution. While HN region contains many OARs, we limited ourselves to the ones that were contoured in the original treatment plans: spinal cord, brain, brainstem, parotid, eye, optic nerve, optic chiasm, and mandible. Not all the OARs were present in all the plans. This usually meant that the dose to the particular OAR was deemed to be too small even before the treatment plan was created to be of concern, or the OAR was part of PTV. We did not collect dosimetric data for “missing” OARs, based on the assumption that the OARs contained in the original treatment plan were the ones that influenced physician's decision to approve the plan.

**Table 3:** Percentage deviation of the various dosimetric parameters describing dose delivered to OARs in homogeneous and bulk density treatment plans from the heterogeneous dose distribution. The dosimetric parameters differing by 2% or more from heterogeneous calculations are shaded in blue.


*Homogeneous Plan*		*#1*	*#2*	*#3*	*#4*	*#5*	*#6*	*#7*	*#8*	*#9*	*#10*
Spinal cord	$D_{\max},\%$	**2.5**	0.8	0.8	**2.1**	1.1	**2.0**	**5.2**	**2.2**	**2.2**	1.4
	$D_{\max}(2\;\text{cc}),\%$	1.6	0.5	0.7	**2.8**	1.3	1.1	−2.5	1.3	1.3	0.9
Brain	$D_{\max},\%$	**2.2**	**2.9**	**3.5**	**2.9**	0.7	**2.0**	**2.5**	**2.7**		
	$D_{\max}\;(2\;\text{cc}),\%$	1.0	**2.4**	**3.7**	**2.4**	1.9	1.4	1.9	**2.3**		
Brain stem	$D_{\max},\%$								**3.9**	**2.0**	0.2
	$D_{\max}\;(2\;\text{cc}),\%$								**2.7**	1.8	1.6
Parotid left	$D_{\mathrm{mean}},\%$	1.1	1.4	0.9	**2.3**	0.4	0.8		1.7	1.0	0.6
Parotid right	$D_{\mathrm{mean}},\%$	**2.7**	1.3	1.0	0.2	1.1	1.2		1.6	1.0	1.2
Eye left	$D_{\max},\%$		−4.6	−1.8	−5.6	−0.1		−2.0			
	$D_{\mathrm{mean}},\%$		−1.2	−1.9	−0.5	0.5		0.3			
Eye right	$D_{\max},\%$		−2.3	−2.7	−0.8	−0.3					
	$D_{\mathrm{mean}},\%$		−0.8	−1.6	−0.7	0.1					
Optic nerve	$D_{\max},\%$							1.6	**5.6**		
Optic chiasm	$D_{\max},\%$							1.1	**2.0**		
Mandible	$D_{\max},\%$										0.5
	$D_{\max}\;(2\;\text{cc}),\%$										1.7

*Bulk Density Plan*		*#1*	*#2*	*#3*	*#4*	*#5*	*#6*	*#7*	*#8*	*#9*	*#10*
Spinal cord	$D_{\max},\%$	0.3	−0.8	−0.3	0.7	−0.3	0.2	−4.0	0.5	−0.3	−0.1
	$D_{\max}\;(2\;\text{cc}),\%$	0.1	−0.3	−0.2	0.4	0.1	−0.2	−3.0	0.7	0.1	−0.1
Brain	$D_{\max},\%$	0.4	0.0	0.0	0.4	0.5	−1.1	−0.4	−1.1		
	$D_{\max}$ (2cc),%	0.1	−0.1	0.7	0.4	0.7	−0.7	−0.1	−0.4		
Brainstem	$D_{\max},\%$								−0.1	0.6	0.0
	$D_{\max}\;(2\;\text{cc}),\%$								−0.2	0.4	0.4
Parotid left	$D_{\mathrm{mean}},\%$	0.5	0.4	0.3	−0.3	0.0	0.2		0.6	0.2	−0.1
Parotid right	$D_{\mathrm{mean}},\%$	0.8	0.3	0.1	0.2	0.1	0.1		0.7	0.2	0.1
Eye left	$D_{\max},\%$		−1.3	−0.2	−1.5	−0.3		−2.8			
	$D_{\mathrm{mean}},\%$		0.2	−0.3	0.1	0.1		−0.6			
Eye right	$D_{\max},\%$		−0.3	−0.4	−0.4	−0.4					
	$D_{\mathrm{mean}},\%$		0.3	−0.2	0.1	−0.1					
Optic nerve	$D_{\max},\%$							−2.3	1.2		
Optic chiasm	$D_{\max},\%$							−0.5	1.0		
Mandible	$D_{\max},\%$										−0.1
	$D_{\max}(2\;\text{cc}),\%$										−0.1

Trends for OARs were similar to those observed for PTV. For homogeneous calculations, there were dosimetric parameters that deviated by at least 2% in eight out of ten patients. The largest deviations exceeded 5% (spinal cord, eye, optic nerve). Mandible was the only OAR with deviations not exceeding 2% in homogeneous treatment plan. Note that dose to mandible was calculated only for one patient. If more patients were involved, we would probably see deviations above 2%.

**Figure 2:**
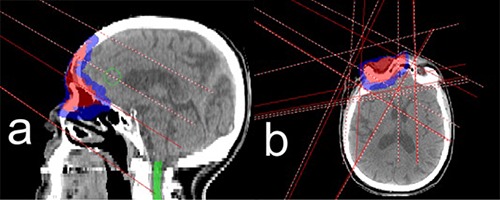
Treatment beams arrangement in sagittal (a) and axial (b) planes for patient #7. The shaded red region is the primary PTV, blue is secondary PTV and green is the spinal cord. There are five treatment beams in total, four are coplanar and one is noncoplanar (couch rotated 90°). Only the noncoplanar beam propagates through the spinal cord. The rest of the beams are superior to the cord and situated in the same axial planes as PTV.

If bulk density was used, then only patient #7 demonstrated deviations exceeding 2%. If this patient's data were omitted, then maximum deviation for bulk density calculations was 1.5%, with most of the deviations being less than 1%. In the case of patient #7, spinal cord, eye, and optic nerve showed deviations larger than 2% when bulk density was used. For the spinal cord, there was only one treatment beam (noncoplanar) propagating through it (see Fig. 2). The rest of the beams were superior to the spinal cord. The attenuation of the noncoplanar beam before it reached the cord was overestimated because it propagated through the volume with tissue density significantly below that of water, but not low enough to consider it air cavity. Since the rest of the beams did not intersect with the spinal cord, the averaging effect of many IMRT beams was absent. As a result, the maximum point dose was underestimated by 4.0%. But, since only one beam propagated through the cord, the absolute dose delivered to the cord was small as compared to the prescription dose. The maximum point dose to the spinal cord calculated using CT‐derived electron density was 14.9 Gy. Maximum dose to $2\;{\text{cm}}^{3}$ was only 0.57 Gy. Similarly, low doses were delivered to the eye and the optic nerve. There were only two beams propagating through each structure. Correspondingly, the maximum point dose to the eye and the optic nerve was only 19.3 Gy and 24.1 Gy, respectively, which was well below the irreversible damage threshold for both organs.

## IV. DISCUSSION

MRI often provides improved contrast resolution between different types of tissues compared to CT. Nevertheless, MRI is usually not used as a stand‐alone radiation therapy simulator due to several technical limitations, one of them being lack of electron density information for treatment planning calculations. Assigning bulk electron density may overcome this problem. Several studies conducted so far demonstrated that the use of bulk density in pelvis and brain is indeed a viable option.^(^ ^9^ ^–^ ^14^ ^)^ The results of this study indicate that with IMRT treatments the same approach can be extended to HN region.

Due to the rather limited effect of heterogeneities in pelvis and brain, simple homogeneous geometry results in acceptable accuracy of dose calculations. HN contains heterogeneities ranging from airways to cortical bone and, as such, is more prone to its effects in dose calculations. Indeed, homogeneous calculations done in this work resulted in dosimetric parameters describing PTV coverage that differed by more than 2% in seven out of ten patients. For OARs, deviations larger than 2% were observed in eight out of ten patients. Therefore, homogeneous geometry is not a valid approach for HN region.

The problem can be corrected by segmenting various tissues and assigning them a bulk density. To improve accuracy, it is preferable to segment as many tissue types as possible and assign them appropriate density. Unfortunately, such approach is not practical. With multiple beams used in IMRT, the impact of various heterogeneities encountered in the HN region on the overall dose is reduced. Hence, high dosimetric accuracy may be achieved by using a limited number of bulk densities. In particular this paper demonstrated that by using just three different density overrides (bone $1.5\;g/{\text{cm}}^{3}$, air cavities $0\;g/{\text{cm}}^{3}$, all other tissue $1\;g/{\text{cm}}^{3}$), one could significantly improve accuracy of dose calculations. All the parameters used in this paper to describe PTV coverage for all ten patients were well within 2% of the CT‐derived density calculations. Dose calculations for OARs had higher deviations because OARs were usually not encompassed by all the beams used in the treatment plan. That increased the effect of the heterogeneities on the overall dose delivered to OARs. Nevertheless, only one patient showed deviations exceeding 2%. The OARs with significant deviation (>2%) were irradiated by only one or two treatment beams in the IMRT plan. Correspondingly, they were located in the low‐dose regions where small differences in absolute dose resulted in higher relative change. But the dose delivered to them was well below the prescription dose, as well as the permanent damage threshold.

Based on the data presented here we conclude that, if bone and air cavities are overridden with proper density, it is feasible to use bulk electron density approach for IMRT treatment planning of HN cancers, which may overcome the problem of the lack of electron density information in MRI images.

## V. CONCLUSIONS

The paper demonstrated that by assigning bulk electron density to bones and air cavities, high dosimetric accuracy could be achieved for dose calculations for HN patients treated with IMRT even in the absence of detailed electron density information provided by CT. This may potentially overcome the problem of the lack of electron density information provided by MRI. As such, it makes the use of MRI as a stand‐alone radiation therapy simulator for HN patients one step closer to reality.
